# Application of *Cinnamomum burmannii* Essential Oil in Promoting Wound Healing

**DOI:** 10.3390/molecules29092080

**Published:** 2024-04-30

**Authors:** Xiangsheng Zhang, Xueyi Lin, Jiayuan Cao, Guofeng Xie, Xinrui Yang, Bingnan Liu, Xin Xu, Fang Cheng, Hongbo Chen, Yuxin Pang

**Affiliations:** 1 School of Traditional Medicine Materials Resource, Guangdong Pharmaceutical University, Yunfu 527325, China; m15257663271@163.com (X.Z.); m13527152763@163.com (X.L.); cjygdpu@163.com (J.C.); lbn0714@163.com (B.L.); 2112148099@gdpu.edu.cn (X.X.); 2 Yunfu Traditional Chinese Medicine Resources and Germplasm Resources Bank Management Center, Yunfu 527399, China; 3 School of Pharmaceutical Sciences (Shenzhen), Sun Yat-sen University, Shenzhen 518107, China; xieguofeng2012@foxmail.com (G.X.); yangxr25@mail2.sysu.edu.cn (X.Y.); 4 College of Pharmaceutical Sciences, Guizhou University of Traditional Chinese Medicine, Guiyang 550025, China

**Keywords:** *Cinnamomum burmannii*, wound healing, macrophages, essential oil

## Abstract

Skin wounds, leading to infections and death, have a huge negative impact on healthcare systems around the world. Antibacterial therapy and the suppression of excessive inflammation help wounds heal. To date, the application of wound dressings, biologics and biomaterials (hydrogels, epidermal growth factor, stem cells, etc.) is limited due to their difficult and expensive preparation process. *Cinnamomum burmannii* (Nees & T. Nees) Blume is an herb in traditional medicine, and its essential oil is rich in D-borneol, with antibacterial and anti-inflammatory effects. However, it is not clear whether *Cinnamomum burmannii* essential oil has the function of promoting wound healing. This study analyzed 32 main components and their relative contents of essential oil using GC-MS. Then, network pharmacology was used to predict the possible targets of this essential oil in wound healing. We first proved this essential oil’s effects in vitro and in vivo. *Cinnamomum burmannii* essential oil could not only promote the proliferation and migration of skin stromal cells, but also promote M2-type polarization of macrophages while inhibiting the expression of pro-inflammatory cytokines. This study explored the possible mechanism by which *Cinnamomum burmannii* essential oil promotes wound healing, providing a cheap and effective strategy for promoting wound healing.

## 1. Introduction

Skin is the body’s largest organ, providing a natural barrier against external damage and exerting a variety of essential protective functions [1]. Wound is the disruption of the functional and anatomical intactness of the epidermis or subcutaneous tissues caused by external physical or chemical influences on the skin [2]. Skin wounds have a significant impact on the global healthcare system. It is calculated that nearly 1 billion people are affected by acute and/or chronic wounds [3,4]. Appropriate medicines and formulations are necessary to prevent wound development and accelerate healing due to the rising medical costs [5].

Skin wounds can be classed as acute or chronic depending on the pathogenesis and consequences [6]. Wound repair is a multi-step process that involves coordinating multiple cell types and signaling molecules [7]. The process can be classified into four distinctive but interlinked stages: hemostasis, inflammation, proliferation and remodeling [7]. Hemostasis begins immediately after injury. Activated platelets form fibrin clots and last from seconds to minutes [8,9]. The inflammatory phase occurs after hemostasis and is characterized by successive infiltration of neutrophils, macrophages and lymphocytes [10]. In particular, factors such as infection can extend this period. An excessive inflammatory reaction interferes with continued wound healing and eventually results in scarring through continued stimulation of fibroblasts. The proliferative stage is primarily controlled by fibroblast and angiogenesis processes and is characterized by the formation of granulation tissue [11]. During wound healing, new blood vessels and fibroblasts are generated, which in turn produce myofibroblasts. The contraction of myofibroblasts leads to a reduction in the wound area. The final stage of wound healing, known as the maturation stage, begins 2–3 weeks after tissue injury. During this stage, the formation of granulation tissue ceases, and collagen remodeling takes place [12].

The switch from the initially inflammatory phase to the proliferative phase is a critical point in determining the outcome of healing, and excessive inflammation is detrimental to wound repair [10,13]. Macrophages are recognized as the primary contributors to the transition from the inflammatory phase to the proliferation phase. They play a critical role in inflammation, fibrosis and wound healing by releasing cytokines and growth factors. There are two major subtypes of macrophages: classically activated M1 macrophages, which have inflammatory properties, and alternative activated M2 macrophages, which have anti-inflammatory properties. Macrophage M1 polarization can be induced by lipopolysaccharide (LPS) through the activation of the major transcription factor nuclear factor κB (NF-κB). Inhibition of the NF-κB pathway is effective in suppressing macrophage M1 polarization [14]. Jian Xie et al. constructed aligned nanofibers. The material was found to enhance wound healing by reducing the pro-inflammatory M1 phenotype, upregulating the pro-healing M2 phenotype and attenuating the local inflammatory reaction of skin wounds [15]. Zhuolong Tu et al. designed bioactive materials with anti-inflammatory, antioxidant, and antibacterial properties and M2 polarization function to provide efficient strategies for wound repair in the treatment of diabetes mellitus [16]. These findings suggest that inhibiting macrophage M1 polarization and the secretion of inflammatory factors contributes to promoting wound healing.

*Cinnamomum burmannii* is a valuable timber tree with aromatic properties that belongs to the Lauraceae family [17]; it is a medicinal plant that has been classified into four chemotypes based on the varying compositions of its essential oil from leaves and bark: cymene/cineol type, cineol type, cineol/borneol type and borneol type [18,19]. The chemical type of the Meipian tree (*Cinnamomum burmannii*) from Meizhou city of Guangdong Province is the borneol type, and its fresh leaves are rich in D-borneol. *Cinnamomum burmannii* is an ancient herb with satisfactory curative effects in China. It contains more than 100 monoterpene and sesquiterpene compounds, including borneol, 1,8-cineol, linalool and cinnamaldehyde [19]. *Cinnamomum burmannii* has been proven to have effects on analgesia, antibacterial activity, antidiabetic properties, antifungal activity, antioxidant activity and antirheumatic properties. The bark powder of *Cinnamomum burmannii* can be used to treat nausea, gastrointestinal upset, cough, chest discomfort, stomach pain and diarrhea [20].

Essential oils (EOs) are products obtained from plants by distillation with water or steam, or by mechanical processes or “dry distillation”, and then by physical separation of the essential oils from the aqueous phase [21]. EOs have been extensively studied for their treatment potential in a variety of diseases [14,22]. Studies indicate that essential oils have a positive effect on cardiovascular disease, respiratory disease, and wound healing [23,24,25]. The essential oil of *Bursera morelensis* has been found to promote wound healing in mice by stimulating the migration of fibroblast cells to the wound site, making them actively involved in collagen production and promoting collagen remodeling [26]. The chemical diversity of essential oils and their ability to interact with various biological targets at the cellular and multicellular levels account for their diverse pharmacological properties. As a result, essential oils are a promising source for the development of new drugs [27]. However, there have been no reports about the effects of *Cinnamomum burmannii* essential oils (BEOs) on wound healing.

In this study, GC-MS was used to determine the essential oil components of the *Cinnamomum burmannii* tree, and network pharmacological prediction was used to explore the pharmacological mechanism of BEO. Subsequently, the antibacterial activity of *Cinnamomum burmannii* essential oil on common *Escherichia coli* and *Staphylococcus aureus* on the skin surface was explored. The possible molecular mechanism and application of prune tree essential oil in promoting wound healing were verified firstly in vitro and in vivo. The study found an affordable and readily available treatment for skin wounds and established a foundation for the future development of BEO for use on the skin.

## 2. Results

### 2.1. Chemical Characterization of Essential Oil

**Figure 1:**
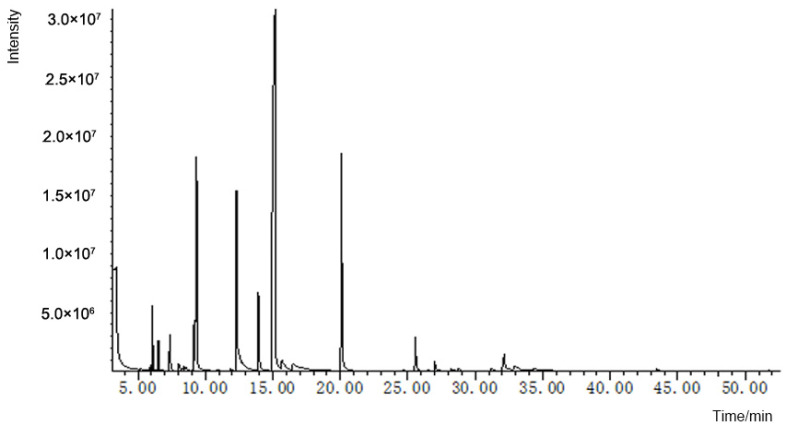
GC-MS analysis of *Cinnamomum burmannii* essential oil total ion flow diagram.

The essential oil extracted from *Cinnamomum burmannii* was analyzed using GC-MS to identify its constituents. The outcomes are presented in Figure 1 and Table 1. The NIST11 standard mass spectrum library identified thirty-two types of chemical compounds with a match degree of over 80%, accounting for 95.44% of the total components in BEO. The main compounds were endo-borneol (43.34%), linalyl propanoate (11.91%), bornyl ester (10.48%) and eucalyptol (8.42%). Other important components were (+)-2-bornanone (3.80%), α-terpineol (2.03%), caryophyllene (1.84%) and terpinen-4-ol (1.69%) (Table 1).

### 2.2. BEO Network and Shared Targets with Wound Healing

**Figure 2:**
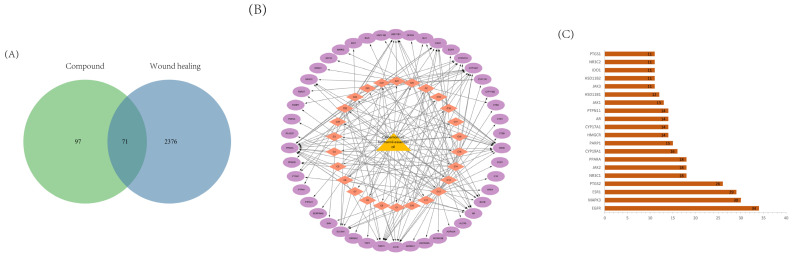

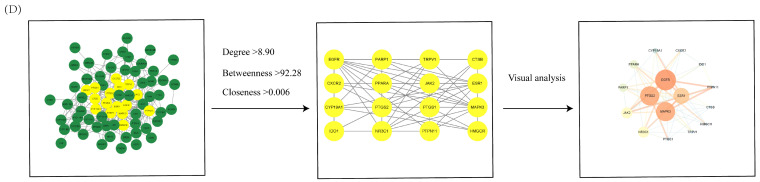

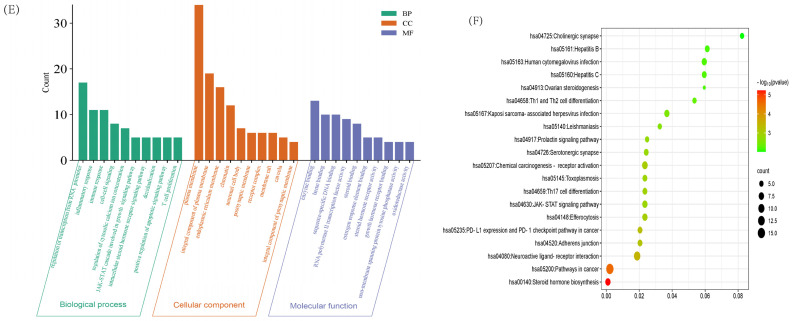
Network pharmacology predicts the possible factors of BEO promoting wound healing. (**A**) Compound–disease targets were intersected using a Venn diagram. (**B**) The herb–component–target network for *Cinnamomum burmannii* essential oil in wound-healing treatment. Yellow: herb; Pink: component; Purple: target. (**C**) The top 20 core genes of the PPI network. (**D**) Protein–protein interaction (PPI) network of intersecting targets. Green: protein target; Yellow: core protein target; Pink: Visual analysis of core protein target interactions. (**E**) GO analysis. (**F**) KEGG analysis. BP: biological process, CC: cellular component, MF: molecular function.

After 32 candidate bioactive ingredients were eliminated, 168 potential targets were obtained from the drug database for further analysis. The 2447 wound-healing-related targets in the database were then compared with the aforementioned 704 potential targets for compounds in BEO, and 71 overlapping targets were found (Figure 2A). The results showed that the BEO has the potential to promote wound healing. A component–target network was constructed based on 71 assumed targets for BEO wound-healing treatment, as shown in Figure 2B.

The above 71 putative targets were analyzed through the STRING database for protein interaction and the network was visualized using Cytoscape (Figure 2D). Then, according to the degree value, the top 20 key targets of the PPI network were identified (Figure 2C). Among them, EGFR, PTGS2, ESR1, MAPK3 and other targets are located in the center of the network and have significant regulatory functions in the PPI network. EGFR can activate the MAPK signaling pathway, causing a downstream cascade reaction to regulate cell proliferation, differentiation and migration. In the biological process analyzed by GO, inflammation and immune response attracted our attention, and too strong inflammation and immune response would delay the wound healing process, suggesting that the BEO may promote wound healing by reducing inflammation (Figure 2E). Pathway enrichment analysis based on the KEGG pathway revealed potential targets associated with the steroid hormone synthesis, efferocytosis and adhesion junction signaling pathways (Figure 2F).

### 2.3. BEO Promoted Wound Healing In Vitro

When the skin is wounded, harmful bacteria such as *Escherichia coli* and *Staphylococcus aureus* can easily invade the tissues, causing infection and tissue damage [28]. We revealed the essential oil inhibited bacteria and fungi to varying degrees. Table 2 shows the bacteriostatic zone morphology and area of BEO against *E. coli* and *S. aureus*. The results showed that the MIC of BEO against *E. coli* and *S. aureus* was 16 mg/mL, and the MBC against *E. coli* and *S. aureus* was 32 mg/mL.

**Figure 3:**
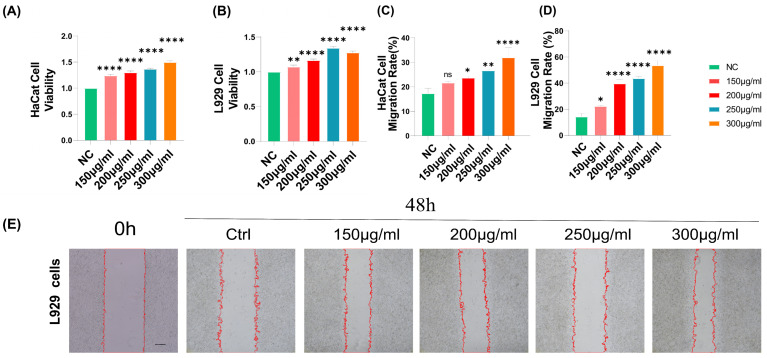

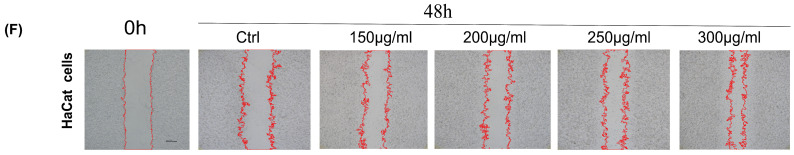
Effects of BEO on fibroblasts and epithelial cells in vitro. (**A**,**B**) The proliferation effect of BEO on HaCaT and L929 cells was determined by CCK8. Wound scratch assay of L929 cells and HaCaT cells, which were incubated with PBS (Ctrl), 150 μg/mL, 200 μg/mL, 250 μg/mL and 300 μg/mL BEO for 48 h. Migration rate (**C**,**D**) and representative scratch images (**E**,**F**) of each group are shown. Scale bar: 100 μm. *n* = 3. *, *p* < 0.05; **, *p* < 0.01; ****, *p* < 0.0001; ns, not significant.

During wound healing, neighboring stromal cells are required to migrate to the wound site and then proliferate. The effect of proliferation was studied by the CCK8 activity assay. The results showed that BEO had a positive concentration correlation for promoting the proliferation of HaCaT cells when the concentration was 100–300 μg/mL. The proliferation of HaCaT cells reached a maximum of 149% when the BEO concentration was 300 μg/mL, and the proliferation of L929 cells reached a maximum of 134% when the BEO concentration was 250 μg/ mL (Figure 3A,B). In vitro scratch wound assays on HaCaT and L929 monolayers were used to evaluate the effect of the extracts on skin cell migration. The results showed that BEO levels at 150, 200, 250 and 300 μg/mL could promote the migration of HaCaT cells and L929 cells (Figure 3C–F).

### 2.4. BEO Attenuated Inflammatory Response In Vitro

**Figure 4:**
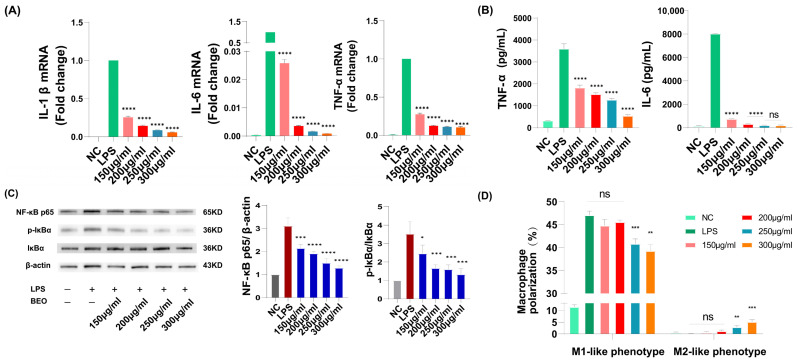

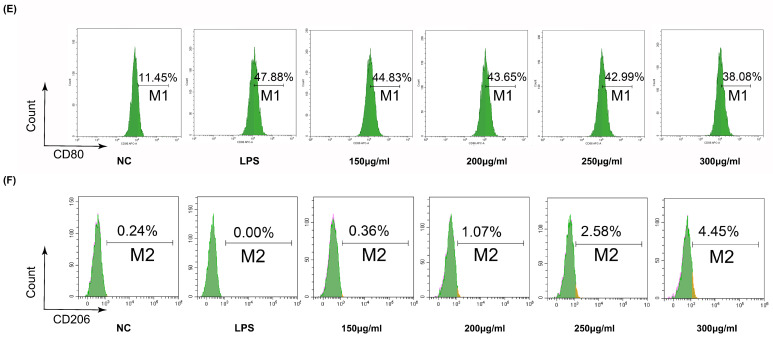
BEO attenuated the inflammatory response in RAW 264.7 cells stimulated by LPS. (**A**) qPCR of IL-1β, IL-6 and TNF-α levels in RAW 264.7 cells that were stimulated by LPS at 24 h from different groups. (**B**) The level of IL-6 and TNF-α secreted by RAW 264.7 cells treated with BEO was determined by ELISA. (**C**) Effects of 150 μg/mL, 200 μg/mL, 250 μg/mL and 300 μg/mL BEO on the expression of NF-κB/p-IκBα protein. (**D**) The cell typing of RAW 264.7 was detected by flow cytometry. Polarization rate (**D**) and sectional images (**E**,**F**) of each group are shown. *n* = 3. *, *p* < 0.05; **, *p* < 0.01; ***, *p* < 0.001; **** *p* < 0.0001; ns, not significant.

Excessive inflammation can disrupt tissue homeostasis and hinder wound healing. Macrophages and secreted cytokines play an important role in the wound-healing process. To simulate the excessive inflammation caused by macrophages cell line, RAW 264.7 cells were treated with LPS, and the expression of inflammatory factors including IL-1β, IL-6 and TNF-α was detected by q-PCR. The results showed that the mRNA expression levels of IL-1β, IL-6 and TNF-α were significantly increased after oil incubation (Figure 4A). BEO had a more significant effect on reducing the secretion of inflammatory cytokines in macrophages than the positive drug epidermal growth factor (EGF). In addition, the secretion of IL-6 and TNF-α was significantly decreased in a dose-dependent manner (Figure 4B).

Macrophage polarization is involved in many signaling pathways, such as the NF-κB, JAK2/STAT3, ROS/ERK and mTOR signaling pathways [29]. Western blot analysis was performed to detect the expression of related proteins in RAW 264.7 cells treated with LPS. The results demonstrated that the essential oil significantly reduced the levels of NF-κB p65 and p-IκBα in a dose-dependent manner after LPS activation (Figure 4C). Flow cytometry was utilized to analyze the effects of different concentrations of BEO on the polarization of macrophages into M1 and M2 phenotypes. The findings indicated that at concentrations of 150 and 200 μg/mL, there was no significant difference observed in the inhibition of M1 polarization or the promotion of M2 polarization in macrophages (Figure 4E,F). However, at concentrations of 250 and 300 μg/mL, BEO exhibited inhibitory effects on M1 polarization and promoted M2 polarization in macrophages. In comparison to the LPS-stimulated group, BEO concentrations of 250 and 300 μg/mL reduced the polarization of M1 macrophages by 4.89% and 9.8%, respectively, while increasing the polarization of M2 macrophages by 2.3% and 4.5%, respectively (Figure 4D).

### 2.5. BEO Promoted Wound Healing In Vivo

**Figure 5:**
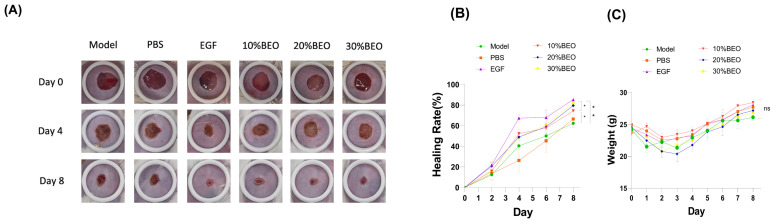

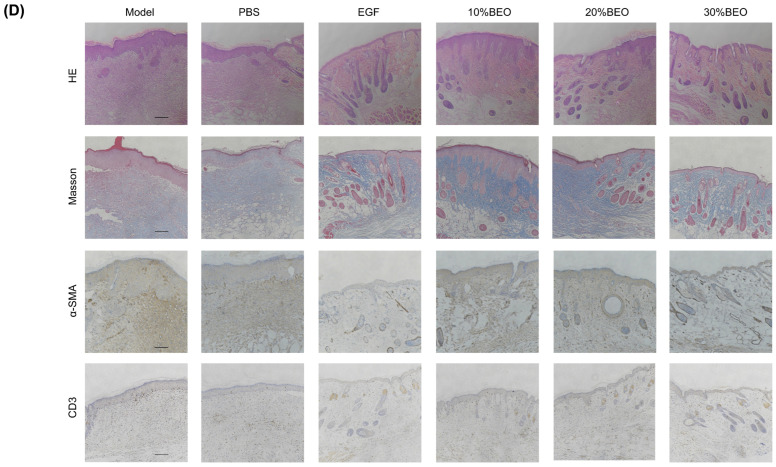
BEO accelerates skin wound healing in mice. (**A**) Representative images of skin wounds of mice treated by PBS, EGF, 10% BEO, 20% BEO and 20% BEO after 8 days. (**B**,**C**) PBS, EGF, 10% BEO, 20% BEO, and 20% BEO groups. *n* = 3. (**D**) Representative images of the healed skin area on day 8 with hematoxylin–eosin (HE), Masson trichromatic staining and immunohistochemistry of α-SMA and CD3 antibodies; scale bar: 200 μm. *, *p* < 0.05; **, *p* < 0.01; ns, not significant.

The effect was proved in vitro, but its mechanism of action in vivo remained unclear. To investigate this further, experiments were conducted on skin-cut mice. Medication was administered on the third day after molding to assess the effect of BEO on incision wounds in vivo. Different concentrations of BEO were applied to surgical wounds in mice and compared with the control group (wound without any treatment), positive control group (EGF) and negative control group (PBS). The effect was verified by the change in wound area (Figure 5A). Compared to the healing rate of the control group (62.39%), the wound-healing rates of 10%BEO (74.37%), 20%BEO (79.51%) and 30%BEO (81.72%) were all higher.

The HE staining results showed that the skin structure of the EGF group and 30%BEO group was more complete, the epidermal layer was thinner, keratinization was improved, and hair follicles and glands were formed compared with the model group (Figure 5D). The collagen fibers (blue) in the EGF group and 30%BEO group were more abundant and more orderly than those in other groups, as determined by Masson staining (Figure 5D). The α-SMA immunohistochemistry results showed that the expression of α-SMA (brown) was significantly up-regulated in the model group and the negative control group compared with the EGF group and the BEO group at different concentrations, as shown in Figure 5D. BEO reduces the infiltration of CD3+ T cells in the local microenvironment at the wound site, which is consistent with the wound-healing process (Figure 5D).

### 2.6. BEO Reduces the Levels of Inflammatory Factors in the Healing Skin

**Figure 6:**
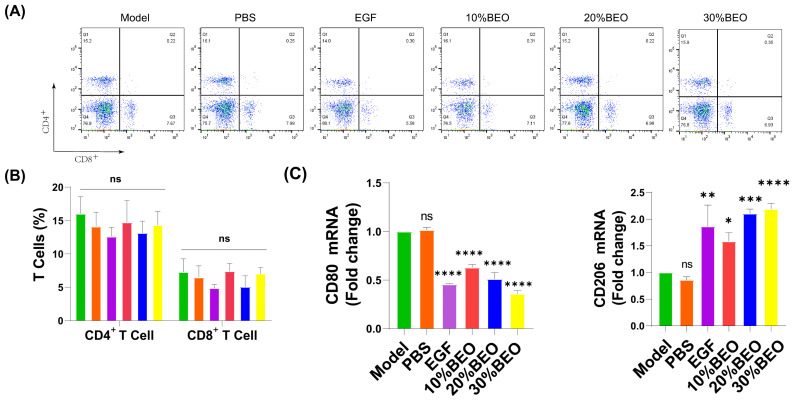

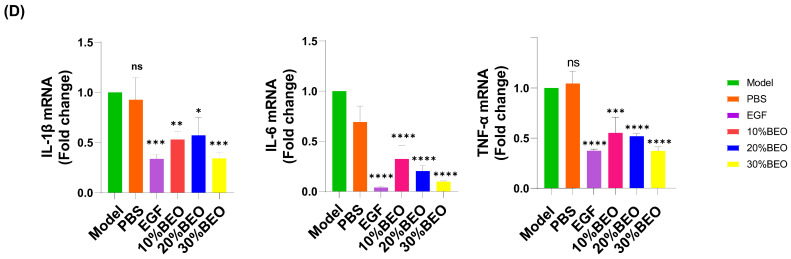
BEO reduces inflammatory factor expression, as well as promoting CD206 mRNA expression. (**A**,**B**) The types of spleen T cells were detected by flow cytometry. (**C**) q-PCR levels of CD-80 and CD-206 in each group on day 8 of the wound surface. (**D**) q-PCR levels of IL-1β, IL-6, and TNF-α in each group on day 8 of the wound surface. *, *p* < 0.05; **, *p* < 0.01; ***, *p* < 0.001; ****, *p* < 0.0001; ns, not significant.

The mRNA levels of inflammatory factors TNF-α, IL-1β and IL-6 in the local wound microenvironment were detected, and TNF-α and IL-1β were significantly decreased, especially in the 30%BEO group (Figure 6D). In addition, compared with the model group, the expression of CD80 in the BEO-treated group was reduced, while CD206 was increased (Figure 6C), which was consistent with the trend of the wound-healing rate. Above all, BEO played a role in resisting external infection, reducing inflammation levels and promoting wound healing. Flow staining typing of lymphocytes isolated from the spleen of mice showed that there was no significant difference in the positive rates of CD4+ T cells and CD8+ T cells among all groups (Figure 6A,B), indicating that the local application of BEO had no significant toxicity.

## 3. Discussion

Currently available drugs for wound healing are often expensive and difficult to prepare. BEO, which is enriched with dextrose and has antimicrobial and anti-inflammatory properties, has the potential to be a more cost-effective and easily accessible wound healing treatment. In this study, we determined the main components of BEO by gas chromatography–mass spectrometry. The results showed that there were 32 chemical components in BEO, and borneol was the main component, which was consistent with the results of Liu Xiaomin et al. [30]. In addition, our essential oil contained lower levels of caryophyllene and bornyl acetate and higher levels of linalyl propanoate, bornyl ester and eucalyptol than theirs. Among these components contained in BEO, it was predicted by the STITCH database that α-terpineol might promote fibroblast proliferation, which was experimentally verified by Smiljanić K et al. [31]. Eucalyptol has antioxidant and anti-inflammatory effects, and eucalyptol-loaded nanoemulgel can accelerate wound healing [32], while borneol has antibacterial and anti-inflammatory effects, and BEO’s ability to promote wound healing may be related to these components. In addition, we selected phytochemicals in the essential oil of *Cinnamomum burmaannii* that may be related to the promotion of wound healing, analyzed their pharmacological effects and compared them with other cinnamon varieties (*Cinnamomum cassia*, *Cinnamomum verum* and *Cinnamomum loureiroi*), and the specific results are shown in Table 3. Moreover, we also found that BEO could inhibit *Escherichia coli* and *Staphylococcus aureus*.

**Figure 7:**
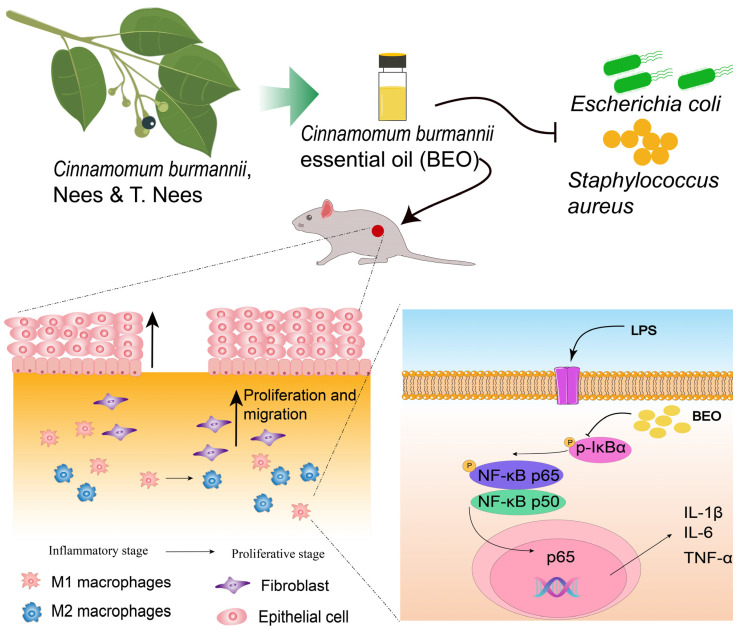
Graphical abstract: mechanism by which *Cinnamomum burmannii* essential oils promote wound healing.

Our network pharmacology results suggested that BEO may promote wound healing by stimulating cell proliferation and migration, as well as modulating inflammatory and immune responses. We subsequently performed experiments to test this hypothesis in vitro and in vivo. Besides promoting skin stromal cell proliferation and migration, BEO significantly reduced the mRNA expression levels of IL-1β, IL-6, and TNF-α through the NF-κB pathway in RAW 264.7 cells. Additionally, the flow results showed that BEO could inhibit macrophage M1 polarization and promote M2 polarization. In animal experiments, the effect of BEO on wound healing in mice was studied. BEO with a 30% concentration had the best healing effect. Histological analysis of skin tissues revealed that the skin structure was more complete and collagen fibers were more regularly arranged in the treated group than in the model group. Additionally, the expression levels of inflammatory factors TNF-α, IL-1β and IL-6 in the local wound microenvironment of the treatment group were reduced, which was consistent with the wound-healing process. Excessive inflammatory factors during wound healing could lead to scar formation and slow wound healing in later stages [40], and the results suggested BEO may also reduce scar formation during wound healing. The possible mechanism by which BEO promotes wound healing is shown in Figure 7.

Our study demonstrated that BEO promotes skin wound healing in mice and explored some possible mechanisms, which provided some basis for the future development of this essential oil for other applications. However, this study had some limitations. We did not investigate the individual essential oil ingredients to validate their specific roles, and further research is needed.

## 4. Materials and Methods

### 4.1. Materials

*Cinnamomum burmannii* essential oil (BEO) was provided by huaqingyuan Biotechnology Co., Ltd. (Meizhou, China). Other materials used were Dulbecco’s modified Eagle medium (Thermo fisher, Waltham, MA, USA), penicillin/streptomycin (Gibco, Waltham, MA, USA), and fetal bovine serum (EallBio, Beijing, China). Monoclonal antibodies against β-actin, and NF-κB p65 were purchased from MedChemExpress Inc. (MCE, Monmouth Junction, NJ, USA). Epidermal growth factors (Sigma–Aldrich, St. Louis, MO, USA), RNA isolater Total RNA Extraction (Vazyme, Nanjing, China), lipopolysaccharide (LPS) (*Escherichia coli*, serotype 0111:B4) and all other chemicals were obtained from Sigma Chemicals (St Louis, MO, USA).

### 4.2. Gas Chromatography–Mass Spectrometry (GC-MS) Characterization of BEO

The essential oil from *Cinnamomum burmannii* was characterized by gas chromatography–mass spectrometry (GC-MS). The GC-MS system used was an Agilent Technologies gas chromatograph (model 7890A) (Agilent, Santa Clara, CA, USA) linked to a mass spectrometer (model 5975C) (Agilent, Santa Clara, CA, USA) fitted with an HP-5MS column (30.0 m × 250 μm i.d. and 0.25 μm film thickness). The sample was injected by split, using 1 μL of BEO. The experiment began at a starting temperature of 50 °C and then increased to 145 °C at a rate of 3 °C/min. It then rose to 250 °C at a rate of 5 °C/min. The gasification chamber temperature was maintained at 240 °C, while the transmission line temperature was kept at 250 °C. The carrier gas used was helium with a flow rate of 1.0 mL/min. Qualitative analysis was performed using the MS database NIST11 and retention time to identify the detected components. The screening results of the database should exclude the column loss peak.

Quantitative analysis: The area-normalized quantitative method expresses the quantitative result as the ratio of the peak area of the determined component to the sum of the areas of all determined components. The following formula is used for this method.

$$C_{i}=\frac{Ai}{A1+A2+A3+\ldots Ai+\ldots An}\times 100\%$$

where the following definitions hold: *C*_i_—the content of an identified ingredient, %; Ai—the peak area of an identified component; *n*—the total number of identified components.

### 4.3. Bacterial Strains

*Escherichia coli* ATCC 25922 (*E. coli*) and *Staphylococcus aureus* (*S. aureus*) ATCC 25923 were both obtained from the Industrial Microbial Culture Collection and Management Center of China and routinely cultured on Luria-Bertani (LB) liquid medium.

### 4.4. Antimicrobial Activity of the BEO

The antibacterial activity of the essential oil was determined by the disc diffusion method. The medium for microbial growth was LB solid medium. The medium was sterilized at 110 kPa pressure in an autoclave at 121 °C for 20 min. Then, 8 mL of LB solid medium was added into a 10 mL dish, and after the medium cooled completely, 0.1 mL bacteria was inoculated with a McFarland ratio of 0.5. The sterile test strip (diameter 6 mm) was placed on the surface of the inoculation medium and impregnated with 60 μL essential oil solution at the concentrations of 10 mg/mL, 30 mg/mL, 50 mg/mL and 70 mg/mL (1:10 *v*/*v*, 10% ethanol). The samples were incubated at 37 °C for 24 h. The diameter of the inhibition zone was measured after incubation. The presence of a bacteriostatic zone indicated that the essential oil had bacteriostatic activity. The solvent 10% ethanol was used as the negative control.

### 4.5. MIC and MBC

The microdilution method was used to determine the minimum inhibitory concentration (MIC) and minimum bactericidal concentration (MBC) of the essential oil. The essential oil was dissolved in 10% ethanol and then diluted in MHB medium with concentrations ranging from 2 μg/mL to 256 μg/mL. A 96-well microporous plate was used to carry out the microdilution experiment, and 180 μL of each essential oil solution and 20 μL of each bacterium with a 0.5 McFarland ratio were taken. The negative control was treated with MHB medium supplemented with 10% ethanol. The 96-well plates were incubated under a bacterial incubator for 24 h. The absorbance at 600 nm was then measured. The MIC is the lowest essential oil concentration with 100% inhibition of bacterial growth. To determine the MBC, 100 μL was removed from a hole without bacterial growth and transferred to a Petri dish (100 mm diameter) containing LB solid medium (15 g/L Agar) that was then stored in a laboratory oven at 37 ± 2 °C for 24 h. Concentrations of essential oils that did not show bacterial growth after 24 h of incubation were considered fungicides.

### 4.6. Network Construction and Prediction of Genes Associated with Wound Healing

The chemical composition of the input was determined through GC-MS analysis via Pub Chem (https://pubchem.ncbi.nlm.nih.gov/ (accessed on 15 November 2023)), and then Swiss Target Prediction (http://www.swisstargetprediction.ch (accessed on 15 November 2023)) was applied to predict targets based on the primer obtained. Finally, wound-healing protein targets were retrieved from the OMIM (https://www.omim.org/ (accessed on 15 November 2023)), Gene Cards (https://www.genecards.org (accessed on 15 November 2023)) and CTD (http://ctdbase.org/ (accessed on 15 November 2023)) databases. After eliminating duplicate genes, we analyzed the two genes to identify potential targets. Next, we input these targets into STRING (https://string-db.org/ (accessed on 11 January 2024)) to obtain relevant details on protein interactions. We then created a protein–protein interaction (PPI) network using Cytoscape. Subsequently, we enriched the candidate targets using Gene Ontology (GO) and Kyoto Encyclopedia of Genes and Genomes (KEGG) pathways with Metscape (https://metascape.org/ (accessed on 11 January 2024)).

### 4.7. Cell Culture

RAW 264.7 cells, L929 cells (L929 mouse fibroblast cells) and HaCaT cells (human keratinocyte cell line) were cultured in Dulbecco’s modified eagle medium (DMEM, Gibco, Waltham, MA, USA) supplemented with 10% (*v*/*v*) fetal bovine serum and 1% (*v*/*v*) penicillin/streptomycin (P/S, Gibco, Waltham, MA, USA). When the cells grew to 80~90% density, the cells were washed with PBS and then digested with 0.25% pancreatic enzyme for centrifugation, and the RAW 264.7 cells did not need pancreatic enzyme for digestion.

### 4.8. In Vitro Cell Viability and Cytotoxicity Test

The cell viability and cytotoxicity of BEO were evaluated in different skin cell lines (HaCaT and L929) by cell counting kit-8 (CCK8) assay. Cell suspensions with a density of 5 × 10^5^ cells /mL were uniformly inoculated on 96-well plates with 100 μL per well and then placed in incubators for overnight culture, and the old medium was removed. Then, 100 μL of fresh medium was added to the control group, and 100 μL of medium mixed with the drug solution was added to the administration hole, and the culture was continued for 24 h. The medium was removed, 100 μL of CCK-8 reagent was added to each well, and the wells were incubated at 37 °C for 1~2 h away from light. Absorptivity (OD) at 450 nm was determined by enzyme labeling.

### 4.9. Scratch Assay

When L929 cells or HaCaT cells reached 90% confluence in 6-well plates, we used a 200 μL pipette tip to vertically scratch the monolayer. Three scratches were repeated in each well, and detached cells were gently washed with PBS to form three clean and straight lines in one direction.

Then, cells were replenished with 10% DMEM, and each well was supplemented with 100 μL PBS (Ctrl), 150 μg/mL BEO, 200 μg/mL BEO, 250 μg/mL BEO or 300 μg/mL BEO. In addition, each well was supplemented with 200 μL of culture medium with cultured peripheral blood mononuclear cells to simulate the immune environment. After 24 h or 48 h, images were captured with a light microscope to record the cells’ migration. Image J 1.54d software was used for scratch area measurement. The calculation formula for the migration rate was as follows:

$$Migration\;Rate(\%)=\frac{{Scratch\;Area}_{t=0}-{Scratch\;Area}_{t=n}}{{Scratch\;Area}_{t=0}}\times 100\%$$

### 4.10. Quantitative Real-Time PCR

The total RNA of RAW 264.7 cells was extracted with TRIzol^®^ (Vazyme, Nanjing, China) reagent and reverse transcribed into complementary DNA (cDNA) with EasyScript^®^All-in-One SuperMix (Trans Gen, Beijing, China), and qPCR was performed with a thermal cycler, using PerfectStart™SYBR Green I (Vazyme, Nanjing, China) as the fluorophore. Next, mRNA levels of IL-1β, IL-6 and TNF-α were detected by qPCR. The primers we used are listed in Table 4.

### 4.11. Enzyme-Linked Immunosorbent Assay (ELISA) Measurements

After 24 h of cell administration, the cell supernatant was placed into a new tube. Culture medium samples were stored at −80 °C until ELISA detection. Using a mouse IL-6 and TNF-α specific ELISA kit, according to the manufacturer’s instructions, concentrations of IL-6 and TNF-α were measured three times in each independent experiment.

### 4.12. Flow Cytometry

After treatment, the cells were removed from the culture plates and washed in a centrifuge tube. First, 100 μL of CD16/32 (1:1000 dilution) was added to block the non-specific binding antibody. Then, 100 μL of APC-coupled anti-CD80 antibody (1:1000 dilution) and Brilliant Violet 421-coupled anti-CD206 antibody (1:1000 dilution) were double-stained at 37 °C in the dark for 20 min. After two steps of PBS washing, APC and Brilliant Violet 421 fluorophores were detected by a flow cytometry system (Beckman Coulter, Brea, CA, USA). For each sample, 3000 events were recorded.

### 4.13. Western Blot

First, RAW 264.7 cells were cultured in 10% DMEM containing different concentrations of BEO (150 μg/mL, 200 μg/mL, 250 μg/mL, 250 μg/mL and 300 μg/mL BEO) for 24 h. The cells were lysed with RIPA lysis buffer supplemented with protease inhibitor cocktail tablets, iced for 30 min and quantified at equal protein weight. Protein samples were separated by 8% sodium dodecyl sulfate–polyacrylamide gel electrophoresis (SDS-PAGE) and transferred to polyvinylidene fluoride (PVDF) membranes. Each PVDF membrane was blocked by 5% skim milk for 2 h, separated and incubated with NF-κB p65, p-IKB α, IKB α, β-actin primary antibody and corresponding secondary antibody at 4 °C overnight. Protein bands were displayed using an ECL chemiluminescence kit. Image J software was used to quantitatively analyze protein bands.

### 4.14. Animal Models and Drug Administration

BALB/c male mice, aged 6 weeks, were obtained from Shenzhen Maike Biotechnology Co., LTD, Shenzhen, China. They were kept in a controlled environment with a 12 h light/dark cycle, at a temperature of 20–24 °C and a humidity of 45–55%. The animal experiment protocols were approved by the Sun Yat-sen University Animal Ethics Committee in China (SYSU-IACUC-2024-000237).

The animal experiment design was as follows: after one week of acclimatization, mice were randomly divided into 6 groups (*n* = 3); the model group; PBS group; EGF group; and 10%BEO, 20%BEO and 30%BEO (*v*/*v*) treatment groups. An appropriate amount of 1% pentobarbital was injected intraperitoneally for anesthesia, and then hair shaving and hair removal were performed on the lower left side of the back spine. The skin was perforated with an 8 mm skin punch to the depth of the fascia layer to establish a uniform skin incision.

The wound site was treated once a day on the 3rd, 4th and 5th days after modeling. The weight changes of the mice were recorded every day, and the wounds of the mice were photographed every other day. The drug was administered on the third day after modeling, every 24 h, and the administration continued for three days. The weight changes of the mice were recorded every day, and the wounds of the mice were photographed every other day (with a circular control ring as a reference); then, Image J was used. The software analyzed the wound healing. On the 9th day, the mice were dissected, the wound-healing skin tissue was cut for RNA extraction and pathological analysis and the spleens of the mice were taken to analyze the typing of T cells.

### 4.15. Histology and Immunohistochemistry Analysis

Briefly, skin tissue samples were immersed in 4% paraformaldehyde overnight, dehydrated with alcohol, cleared with xylene and then embedded with paraffin wax and cut into 5 μm thick paraffin sections. Paraffin sections were stained with hematoxylin (H&E) and Masson trichrome staining, embedded in paraffin and cut into 5 μm thick paraffin sections. Paraffin sections were degummed, rehydrated and subjected to heat-induced epitope extraction for immunohistochemical analysis. Then, they were incubated with primary antibodies of CD3 and alpha smooth muscle (α-SMA) and corresponding secondary antibodies. Slides were used for light microscopy with 100× magnification for observation, and three random fields of view were captured.

### 4.16. Flow Cytometry Was Used to Detect the Activation of Spleen T Cells

The spleen was fully ground on a screen, the screen was rinsed with 1 mL PBS, the screen was rinsed repeatedly for more than 5 times, the centrifuge tube containing cell suspension was centrifuged, the single-cell precipitation of the spleen was supplemented with 2 mL red cell lysate, and the spleen was lysed on ice for 5 min and then centrifuged at 1500× *g*. Then, 100 μL cell stain buffer, 1 μL APC anti-mouse CD4 antibody, 1 μL Brilliant Violet 421TM anti-mouse CD8a antibody and 1 μL FITC anti-mouse CD3 antibody were added to the cell precipitation. After blowing and mixing, the cell precipitation was incubated in ice for 15 min under dark light and then centrifuged at 1500× *g*, 4 °C, for 5 min. The activation of CD4+ and CD8+ T cells in splenic cell suspension was measured by flow cytometry.

### 4.17. Statistical Analysis

The mean ± standard error of the mean (SEM) was determined for all treatment groups and analyzed by GraphPad Prism 7 (GraphPad Software Inc., La Jolla, CA, USA). Each datum is the average of at least three replicates for each group. Statistical analysis was conducted using a *t*-test and one-way analysis of variance (ANOVA) by Tukey’s test. *p* < 0.05 indicates a significant difference (*, *p* < 0.05; **, *p* < 0.01; ***, *p* < 0.001; ns, not significant).

## 5. Conclusions

In this study, we investigated the anti-inflammatory, antibacterial and other effects of BEO and confirmed its role in promoting wound healing through in vivo and in vitro experiments. Furthermore, we found that essential oil exhibits a certain antibacterial effect, which is advantageous due to its complex composition that makes it less prone to causing drug resistance. However, there are certain limitations in this study, such as the identification of the specific component of essential oil responsible for promoting cell proliferation and migration, as well as the underlying mechanism. In future research, it would be valuable to explore the potential application of this essential oil in treating various skin diseases including cuts, burns, diabetic feet, ulcers and infectious wounds. Overall, our findings suggest that this essential oil holds promise as a therapeutic agent for promoting tissue repair and regeneration.

## Figures and Tables

**Table 1:** Chemical composition of *Cinnamomum burmannii* essential oil.

Number	Rt (min)	Abundance (%)	Compound	SI (%)
1	3.2	0.53	Propylene Glycol	90
2	6.09	1.57	(+)-α-Pinene	96
3	6.52	0.86	Camphene	97
4	7.34	0.59	β-Thujene	93
5	7.4	1.19	β-Pinene	97
6	8.02	0.52	β-Myrcene	87
7	8.61	0.26	α-Thujene	87
8	9.16	1.56	o-Cymene	97
9	9.28	1.49	D-Limonene	99
10	9.36	8.42	Eucalyptol	97
11	11.89	0.19	(+)-4-Carene	97
12	12.77	11.91	Linalyl propionate	97
13	13.31	0.28	Linalyl acetate	84
14	13.93	3.8	(+)-2-Bornanone	98
15	15.16	43.34	endo-Borneol	96
16	15.69	1.69	Terpinen-4-ol	96
17	16.59	2.03	α-Terpineol	91
18	20.1	10.48	Isobornyl acetate	99
19	25.56	1.84	Caryophyllene	99
20	27.0	0.67	1,5,9,9-Tetramethyl-1,4,7-cycloundecatriene	98
21	27.28	0.01	Humulene	81
22	28.21	0.13	β-copaene	93
23	28.42	0.13	β-Selinene	99
24	28.78	0.29	Bicyclogermacrene	93
25	30.05	0.03	Cadina-1(10),4-diene	91
26	32.41	0.32	(−)-Spathulenol	91
27	32.92	0.63	Guaiol	99
28	34.2	0.04	(+/−)-Cadinene	89
29	34.36	0.39	1*H*-Cycloprop[e]azulen-7-ol, decahydro-1,1,7-trimethyl-4-methylene-, [1ar-(1aalpha,4aalpha,7beta,7abeta,7balpha)]-	86
30	35.05	0.1	(−)-α-Gurjunene	96
31	35.13	0.06	Oxo-Tremorine	90
32	35.45	0.09	β-Eudesmene	93

**Table 2:** Sensitivity of strains to BEO evaluated using the disc diffusion method and MIC and MBC of BEO.

Microorganisms	Diameters of Inhibition Zone (mm)					MIC	MBC
	EGF	10 mg/mL	30 mg/mL	50 mg/mL	70 mg/mL	BEO (mg/mL)	
*S. aureus*	6.0	6.0	9.6 ± 0.42	11.2 ± 0.28	14.79 ± 0.45	16	32
*E. coli*	6.0	7.34 ± 0.21	8.56 ± 0.53	10.16 ± 0.45	13.8 ± 0.38	16	32

**Table 3:** The phytochemicals and pharmacological activity of various *Cinnamomum* species.

*Cinnamum* Plant	Phytochemicals	Pharmacological Activity
*C. burmaannii*	α-Terpineol Eucalyptol Borneol	Increased fibroblast viability and/or proliferation [33]. Antioxidant and anti-inflammatory [31]. Antibacterial and anti-inflammatory [32].
*C. cassia*	Cinncassiol G and cinnacasol Cinnamaldehyde	Inhibitory effects against proliferation of T cells and B cells [34]. Stimulates angiogenesis, promotes blood circulation [35]. Anti-inflammatory and analgesic [36].
*C. verum*	Cinnamaldehyde benzyl benzoate	Antifungal and antioxidant [37]. Increasing cell proliferation, collagen synthesis, and reepithelialization ratio [38].
*C. loureiroi*	Tannins and saponins	Astringents, healing, antiexudative, anti-irritative, anti-inflammatory, antiseptic, anesthetic and antioxidant [39].

**Table 4:** The primer sequences of qPCR.

Gene	Forward Primer Sequence 5′–3′	Reverse Primer Sequence 5′–3′
Mouse β-actin	GGCTGTATTCCCCTCCATCG	CCAGTTGGTAACAATGCCATGT
Mouse IL-1β	GAAATGCCACCTTT TGACAGTG	TGGATGCTCTCAT CAGGACAG
Mouse IL-6	CTGCAAGAGACT TCCATCCAG	AGTGGTATAGACAGG TCTGTTGG
Mouse TNF-α CD80 CD206	CTGAACTTCGGGGTGATCGG CCTCAAGTTTCCATGTCCAAGGC AAACACAGACTGACCCTTCCC	GGCTTGYCACTCGAA TTTTGAGA GAGGAGAGTTGTAACGGCAAGG GTTAGTGTACCGCACCCTCC

## Data Availability

The original contributions presented in the study are included in the article, further inquiries can be directed to the corresponding author.
